# Collateral Capacity Assessment: Robustness and Interobserver Agreement of Two Grading Scales and Agreement with Quantitative Scoring

**DOI:** 10.1007/s00062-022-01216-4

**Published:** 2022-09-26

**Authors:** Wenjin Yang, Jazba Soomro, Ivo G. H. Jansen, Aashish Venkatesh, Albert J. Yoo, Demetrius Lopes, Ludo F. M. Beenen, Bart J. Emmer, Charles B. L. M. Majoie, Henk A. Marquering

**Affiliations:** 1 Department of Radiology and Nuclear Medicine, Amsterdam University Medical Centers, location AMC, Amsterdam, The Netherlands; 2 Neurointerventional Service, Texas Stroke Institute, Plano, TX USA; 3 NICO.LAB B.V, Amsterdam, The Netherlands; 4 Advocate Aurora Health Brain and Spine Institute, Chicago, IL USA; 5 Department of Biomedical Engineering and Physics, Amsterdam University Medical Centers, location AMC, Amsterdam, The Netherlands

**Keywords:** Intracranial collateral capacity, Automated quantitative score, Manual Tan score, Interobserver agreement, Correlation coefficient

## Abstract

**Background and Purpose:**

Intracranial collateral capacity is conducive to imply parenchymal perfusion of affected territory after acute vessel occlusion. The Tan collateral score is commonly used to assess the intracranial collateral capacity; however, this score is coarsely grained and interobserver agreement is low, which reduces prognostic value and clinical utility. We introduce and evaluate an alternative extended Tan score based on the conventional Tan scale and assess the agreement with a quantitative score.

**Methods:**

We included 100 consecutive patients with a proven acute single large vessel occlusion of the proximal anterior circulation. Collaterals were graded with the conventional and extended Tan score and an automated quantitative score. The extended Tan score is a finer 6‑scale manual score based on the conventional 4‑point Tan scale. The quantitative score is calculated by an automatic software package (StrokeViewer). Interobserver agreement of the manual scores was assessed with the weighted kappa. The Spearman correlation coefficient was calculated to determine the agreement between the manual and automated collateral scores.

**Results:**

The interobserver agreement was higher for the extended score than for the conventional score with a weighted kappa of 0.70 and 0.65, respectively. For the extended and conventional score, the Spearman correlation coefficient for the agreement with the automated score was 0.78 and 0.76, respectively.

**Conclusion:**

Because of the good interobserver agreement and good agreement with quantitative assessment, the extended collateral score is a strong candidate to improve prognostic value of collateral assessment and implementation in clinical practice.

## Introduction

Intracranial collaterals can provide alternative pathways of blood flow in acute ischemic stroke. Collateral scoring based on computed tomography angiography (CTA) allows assessment of parenchymal perfusion of affected territory after acute intracranial large vessel occlusion (LVO) and has been associated with clinical outcome, which is beneficial to decision-making for endovascular treatment (EVT) in acute ischemic stroke [1–7].

The Tan score proposed by Tan in 2009 is commonly used to assess the intracranial collateral capacity on CTA imaging in acute ischemic stroke [8]. As a visual scoring method, it is limited by relatively poor interobserver agreement [9, 10]. The relatively coarse classification of collateralization may be a main reason for this considerable interobserver variability.

Additionally, visual collateral scoring needs experienced readers for consistency. The application of automated and quantitative approaches has the potential to counter this problem. StrokeViewer (Nicolab B. V. Amsterdam, the Netherlands; www.nicolab.com) is a fully automated post-processing software that also allows to quantify patients’ collateral capacity based on single-phase CTA. Previously, it has been shown that this quantitative collateral score is strongly correlated with the Tan score [11].

In this study, we extended the conventional Tan score and compared the correlation between manual and automatic collateral scores. We aimed to explore whether a finer scaled evaluation method of collateral capacity is more reliable with improved interobserver agreement in stroke studies and clinical practice.

## Methods

### Study Participants

The CT angiography (CTA) data were retrospectively collected from 100 consecutive patients from 2 comprehensive stroke centers between January 2018 and November 2020. The scans were performed as part of the stroke protocol at the comprehensive stroke center as well as at several referring regional hospitals. Study-specific inclusion criteria were as follows: age 18 years or older; proximal anterior circulation acute vessel occlusion proven on CTA with single occlusion (carotid bifurcation, ICA‑T, or M1 or M2 segment of the middle cerebral artery, MCA); available baseline single-phase acquisition CTA imaging with thin-slice (< 2 mm) axial images. CTA scans were excluded according to the following criteria: poor image quality such as insufficient brain coverage, extreme movement, or artifacts in the MCA region (e.g. because of the presence of coils). The study was approved by the institutional review boards. Informed written consent from patients was waived related to the retrospective design of the study using anonymized imaging data only.

### Visual Assessment of Collateral Capacity

The collateral capacity on all CTAs was assessed using two visual collateral scores, the conventional Tan score and the extended Tan score. In the 4‑point conventional Tan score scale, a score of 0 refers to absent collateral supply, 1 indicates poor collateral supply (> 0 and ≤ 50%), 2 indicates moderate collateral supply (between 50 and 99%), and 3 points to good collateral supply (with a 100% filling of the MCA territory) [8].

The extended collateral score is a finer grained scale than the conventional Tan score. Similar to the conventional Tan score, the percentage of opacified vessels of the occluded territory is compared to the contralateral hemisphere in the MCA territory. Compared to the conventional Tan score, category 1 is split in 1a and 1b, with a filling of > 0–25 and > 25–50% respectively, and 2 is split in 2a and 2b, with a filling of > 50–75 and > 75–99% of the MCA territory respectively, whilst categories 0 and 3 are the same (see Table 1).

**Table 1:** Overview of manual Tan scores and automated quantitative scores

*Conventional Tan score*		*Extended Tan score*		*Automated quantitative score*
Collateral grade	Filling of the MCA territory (%)	Collateral grade	Filling of the MCA territory (%)	Ratio of vascular volume in the affected and the contralateral side (%)
0	0	0	0	0
1	> 0 and ≤ 50	1a	> 0 and ≤ 25	> 0 and ≤ 25
		1b	> 25 and ≤ 50	> 25 and ≤ 50
2	> 50 and ≤ 99	2a	> 50 and ≤ 75	> 50 and ≤ 75
		2b	> 75 and ≤ 99	> 75 and ≤ 99
3	100	3	100	100

*MCA* middle cerebral artery

Two reviewers, one neurointerventional radiologist and one acute care radiologist both with more than 25 years of experience reviewed all images, blinded to any clinical or radiological information except occlusion side and location. The conventional and extended Tan scores were assessed at separate sessions with at least 3 months between the sessions. A third expert reviewer with more than 15 years of experience resolved any discrepancies between reviewers 1 and 2 to come to a consensus score.

### Automated Quantitative Collateral Score

**Fig. 1:**
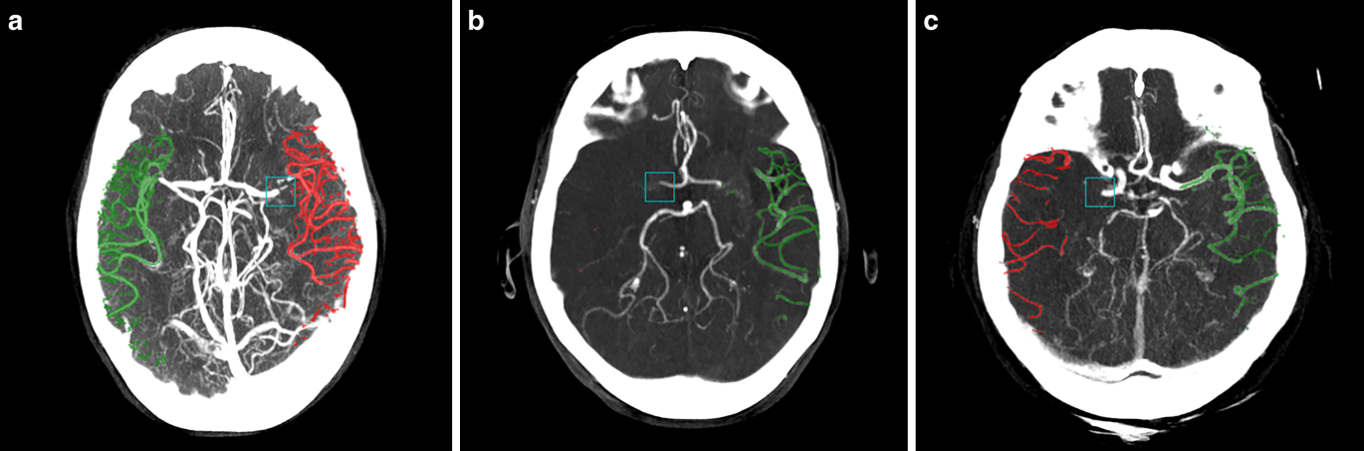
**a** Maximum intensity projection (MIP) of a patient with a left-sided M1 occlusion. Automated collateral score was 93%. Consensus was grade 2 and 2B for the conventional and extended Tan scores, respectively. **b** MIP of patient with right-sided M1 occlusion. Automated score was 1%, consensus was grade 1 and 1A. **c** MIP of a patient with right-sided M1 occlusion. Automated score was 38%, consensus was grade 1 and 1B. The occlusion location as detected by StrokeViewer is depicted with a *blue rectangle*

StrokeViewer was used to automatically quantify collateral capacity. The software has two built-in algorithms for the assessment of both the LVO location and collateral capacity. The LVO location algorithm, which was based on a convolutional neural network (CNN) processed each CTA scan for the presence of an LVO in the anterior circulation (internal carotid artery bifurcation, ICA‑T, M1, or M2 segments of the middle cerebral artery). This process involved cropping the image, registering the brain, predicting the acquisition phase of the scan, and quantifying image characteristics associated with an LVO, resulting in a bounding box around the suspected occlusion location (Fig. 1). The collateral capacity assessment algorithm segmented the vessels downstream of the occlusion and calculated the ratio of the volume of vessels of the affected and the contralateral side. A visual representation of this vessel volume was projected over the CTA (Fig. 1). Additionally, as the automated score was dependent on the occlusion location (visualized bounding box at site of occlusion), both reviewers assessed if the LVO detection by StrokeViewer was acceptable according to their own discretion.

### CTA Acquisition Phase

Due to the possibility of collateral flow underestimation in early arterial CTA scan phases, the scan phase was determined to be included in interpreting the collateral assessment. StrokeViewer features an automatic assessment of the scanning phase and categorized each scan according to a previously described method [11].

### Statistical Analysis

Interobserver reliability analysis was performed by calculating the quadratic weighted kappa statistics for the conventional Tan score and the expanded Tan score. Correlations between the continuous automated and two ordinal Tan scores were assessed by the calculation of the Spearman rank correlation coefficient. Agreement between the continuous and ordinal scores was visualized by plotting boxplots per ordinal scale. To further assess the agreement between the automated quantitative score and the manual scores, the quantitative score was converted into the categories of the conventional and extended Tan score using the thresholds displayed in Table 1. The quadratic weighted kappa analyses were calculated to determine the agreement between the manual score and categorized continuous score after conversion.

Continuous variables were expressed as medians (interquartile range), and categorical variables were expressed as numbers (percentage). All statistical analyses were performed in SPSS (IBM, version 25.0. Armonk, NY, USA).

## Results

Baseline characteristics of the included patients are presented in Table 2. Among all 100 patients, the median age was 69 years (range 60–82 years). The number of females was 52 (52%). The number of left-sided occlusions was 52 (52%). The number of occlusion locations at ICA, M1, and M2 was 28 (28%), 63 (63%), and 9 (9%), respectively.

**Table 2:** Baseline characteristics of the included patients for visual and quantitative collateral scores

	Baseline	
Numbers of patients	100	92
Female—*n* (%)	52 (52)	49 (53)
Age—years, median (IQR)	69 (60–82)	70 (60–82)
Occlusion side		
Left—*n* (%)	52 (52)	47 (51)
Right—*n* (%)	48 (48)	45 (49)
Occlusion location		
ICA—*n* (%)	28 (28)	26 (28)
M1—*n* (%)	63 (63)	61 (66)
M2—*n* (%)	9 (9)	5 (6)

*IQR* interquartile range, *ICA* internal carotid artery

Eight patients were excluded because StrokeViewer incorrectly detected the LVO location. In four of these (all M2 occlusions), no location was given. In three patients (two M1 and one ICA occlusion), the correct side was given but the wrong location, and in one patient (with an ICA occlusion), the wrong side was detected. The latter patient also had a poor contrast load.

Of the 92 scans included for quantitative scores, the number of occlusions on the left side was 47 (51%). The number of occlusions at ICA, M1, and M2 locations was 26 (28%), 61 (66%), and 5 (6%), respectively. Of these scans, 26% were early arterial phase, 32% peak arterial, 30% equilibrium, 9% peak venous and 3% late venous.

### Interobserver Variability of the Manual Scores

The interobserver agreement for the conventional Tan score between observers was substantial with a kappa value of 0.65 (95% confidence interval, CI, 0.42–0.88). The interobserver agreement for the extended Tan score increased to a kappa value of 0.70 (95% CI, 0.59 to 0.81) compared to the conventional Tan score.

### Association Between the Automated Quantitative and Manual Tan Collateral Score

**Fig. 2:**
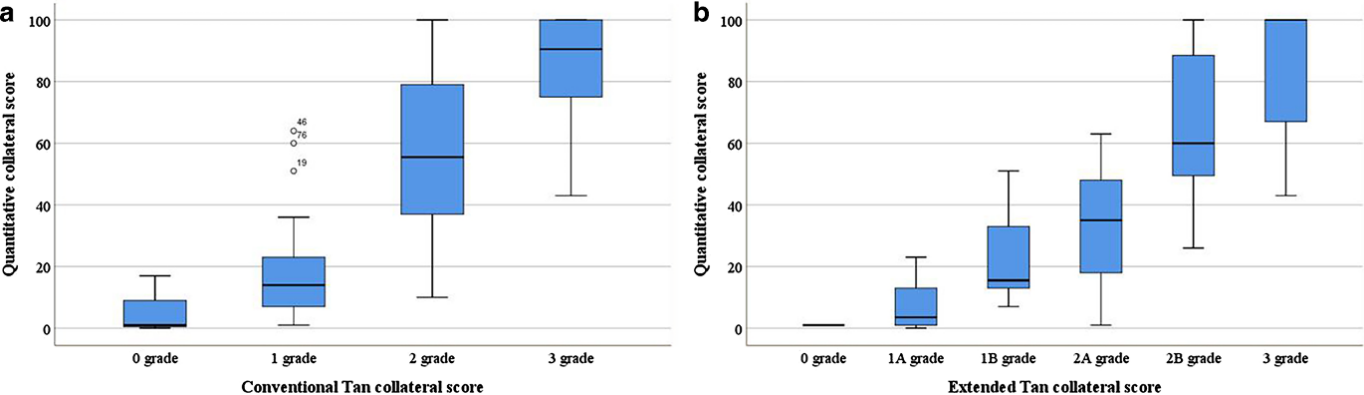
**a** Distribution of the automated collateral score per consensus collateral grade for the conventional Tan score with 4‑point scale, ranging from absent collaterals to good collaterals. **b** Distribution of the automated collateral score per consensus collateral grade for the extended Tan score with 6‑point scale, ranging from absent collaterals to good collaterals

Distribution of the automated collateral score per consensus collateral grade for the conventional Tan score is presented in Fig. 2a. This figure shows a good accordance of visual and quantitative collateral scores. Correlation between the automated score and consensus for the conventional Tan score was strong and statistically significant, with a Spearman ρ of 0.76. The Spearman’s correlation between the automated score and the conventional Tan score as scored by observers 1 and 2 was 0.71 and 0.80, respectively. When turning the automated continuous scores into ordinal variables corresponding to 4‑point scale of conventional Tan score, the kappa value was moderate at 0.59 (95% CI, 0.33 to 0.86) between the automated score and the consensus for conventional Tan score.

Distribution of the automated collateral score per consensus collateral grade for extended Tan score is shown in Fig. 2b. For the extended Tan score, the correlation between the automated score and consensus is stronger than for the conventional Tan score, yielding a Spearman *ρ* of 0.78. The correlation of the automated score with reviewers 1 and 2 was 0.91 and 0.72, respectively. After converting the automated continuous scores into ordinal variables according to 6‑point grading of extended Tan score, the kappa value was 0.67 (95% CI, 0.52 to 0.82).

## Discussion

In our population of 100 patients with an acute ischemic stroke due to an LVO, interobserver agreement of the collateral capacity scoring was substantial for the conventional Tan score and increased further for the extended Tan score. In the 92 cases with correct automated occlusion location detection, the correlation of the automated score with the extended Tan score was stronger compared to its correlation with the conventional Tan score.

The results of our study are in line with previous literature; a post hoc analysis of MR CLEAN also reported strong correlations between the automated collateral score and manual conventional Tan score (Spearman’sρ0.75), and another study with similar interobserver agreement on conventional Tan score (weighted kappa 0.60) [9, 11]. Concerning the correlation between the automated score and the manual score, StrokeViewer was comparable with other automated quantitative tools [11–13].

The conventional Tan score was proposed by Tan in 2009 and is commonly used to assess the intracranial collateral capacity on CTA imaging of these patients with acute ischemic stroke [8]. The association of collaterals appraised by the Tan score with the benefit of endovascular treatment of stroke patients has been proven to be significant, thus making it an important biomarker in the stroke patient selection paradigm [1, 3]. The extended collateral score is a finer grained scale based on the conventional Tan score. Since interobserver agreement is higher for the extended Tan score, this study suggests that it is more reliable to assess the intracranial collateral capacity. In addition, using a more granular Tan collateral score is also possible to be more accurate in predicting treatment outcomes, whereas most of the existing scoring systems for intracranial collaterals cannot reliably predict good clinical outcomes [14]. Of note, more granular scales likely play an essential role in selecting older patients and patients in a late window. For instance, patients over the age of 80 years with collateral score 2b might benefit differently from endovascular treatment than 2a, like the specific inclusion criteria for core infarct threshold and age in the DAWN trial [15]. Thus, we will be intrigued to verify this in MR CLEAN NO-IV substudy. Still, visual collateral scores are relatively challenging to score in an acute stroke setting where time is of the essence. Moreover, specific knowledge is needed for readers to assess collaterals. Artificial intelligence has been applying to quantify collateral flow in an automated fashion in the most recent years [12, 13, 16]. One of these automatic tools, StrokeViewer qualifies collateral score by calculating the ratio of the volume of vessels with the HU values of the affected to the healthy side. Even for the coarse conventional Tan score, correlation with the much more precise, automated assessment method was strong. For the extended Tan score, the strength of the correlation further increased. Because of its consistency, automated scoring may become valuable in acute care stroke situations. This is beneficial in settings where an experienced radiologist is not immediately available, which is often the case in most smaller and rural hospitals, especially off-hours. Nevertheless, it is worth mentioning that reviewers need to visually check the occlusion location identified with the automated score before clinical actions or decisions are taken.

In this study, the collateral capacity was assessed based on single-phase CTA; however, single-phase CTA just acquired collateral flow enhancement on a particular scan phase in a single snapshot, which could lead to an underestimation of collateral capacity in the early artery phase. The multiphase CTA with a better temporal resolution is expected to overcome this limitation [17]. Moreover, single-phase CTA does not contain the time information related to bolus velocities, such as cerebral blood flow (CBF), mean transit time (MTT), and time to peak (TTP) in the CT and MR perfusion. It is well known that the abundance of vasculature and blood velocity are two important aspects of collateral capacity. Notably, in routine clinical practice, a warning is given every time an early phase is detected by StrokeViewer, as part of the intended use of the software. This allows users to incorporate the scan phase into their decision-making process.

This study has some limitations. Patients were collected retrospectively and with a relatively small sample size, although it was ensured that the population was representative of clinical practice. Also, the sensitivity of StrokeViewer LVO detection was reported to be 92% for M2 occlusions compared to 99% for occlusions in the M1 and ICA [18]. Moreover, an incorrect LVO location identification was an exclusion criterion in this study, so M2 patients were relatively often excluded from the collateral score agreement analysis. Still, after the occlusion of the incorrect detected LVO cases, there remained a representative number of patients with an M2 occlusion with successful LVO detection and collateral assessment in the final analysis in this study. Furthermore, the manual collateral scoring was performed by experienced reviewers under a circumstance different from the emergency setting. Finally, this study did not associate clinical outcomes with the various collateral scores addressed in this study, which requires further investigation.

## Conclusion

Interobserver agreement for the extended Tan score with a 6-point scale is good, and the extended collateral score also agrees more strongly with the quantitative collateral score than the conventional one. This study suggests that a finer grained collateral score is a more reliable tool to implement in in clinical research and care.
